# Multidrug-Resistant Burkholderia cepacia, Candida dubliniensis, and Candida glabrata Infected Pancreatic Pseudocyst

**DOI:** 10.7759/cureus.8811

**Published:** 2020-06-24

**Authors:** Sarah Rawi, Bryan Stringer, Myrla Sajo

**Affiliations:** 1 Internal Medicine, University of Connecticut Health Center, Farmington, USA; 2 Internal Medicine/Infectious Disease, Saint Francis Hospital and Medical Center, Hartford, USA

**Keywords:** burkholderia, candida, pancreatitis, resistant, infected, pseudocyst

## Abstract

*Burkholderia cepacia* is a gram-negative bacillus that is most commonly associated with pneumonia in the immunocompromised patients. The most common organisms associated with pancreatic infections are *Escherichia coli*, *Klebsiella pneumoniae*, *Enterobacter *spp*.*, and *Enterococcus *spp. We report a case of a 45-year-old gentleman with recent acute pancreatitis who presented with hypoglycemia, altered mental status, worsening epigastric pain, and early satiety. He was diagnosed with a large peripancreatic infected cyst which grew multidrug-resistant (MDR) *Burkholderia cepacia*, *Candida glabrata*, and *Candida dubliniensis*. This case report focuses on the importance of distinguishing and recognizing risk factors for this MDR organism, in order to provide better patient care.

## Introduction

Pancreatic cysts have up to 13.5% prevalence and are classified between non-neoplastic and neoplastic cysts. Infected pseudocysts are non-neoplastic and are a result of complications of pancreatitis [1,2]. The Atlanta classification has now revised the definition of ‘pseudocyst’ as a complication of interstitial edematous pancreatitis, while ‘walled-off necrosis (WON)’ is used for patients with necrotizing pancreatitis; both arising around four weeks after acute pancreatitis [3]. The most common organisms associated with pancreatic infections, including abscesses, are *Escherichia coli*, *Klebsiella pneumoniae,* *Enterobacter *spp*.*, and *Enterococcus *spp. Rarely, other organisms involved are *Pseudomonas aeruginosa*, *Staphylococcus *spp*.*, *Streptococcus *spp*.*, and *Bacteroides* [4]. *Burkholderia cepacia* is a gram-negative obligate aerobe and has been described as a pathogenic organism in humans with chronic granulomatous disease [5]. It is also frequently described in patients with cystic fibrosis and has been associated with a worse prognosis in those who are colonized [1,6]. *B. cepacia* is known to be susceptible to trimethoprim/sulfamethoxazole (TMP-SMX), ceftazidime, carbapenems, ureidopenicillins, fluroquinolones, minocycline, and chloramphenicol [7]. Antifungal agents commonly used against *Candida *species include echinocandins, amphotericin B, and azoles [8]. Although frequently associated with immunocompromised hosts, *Burkholderia* species are emerging pathogens in healthcare settings, with cases of urinary tract infections, pneumonia, and septic arthritis sited in the literature [1,7]. Thus, it is essential to continue investigating risk factors for this infection in different patient populations and to pay close attention to the cases that emerge. 

## Case presentation

**Figure 1:**
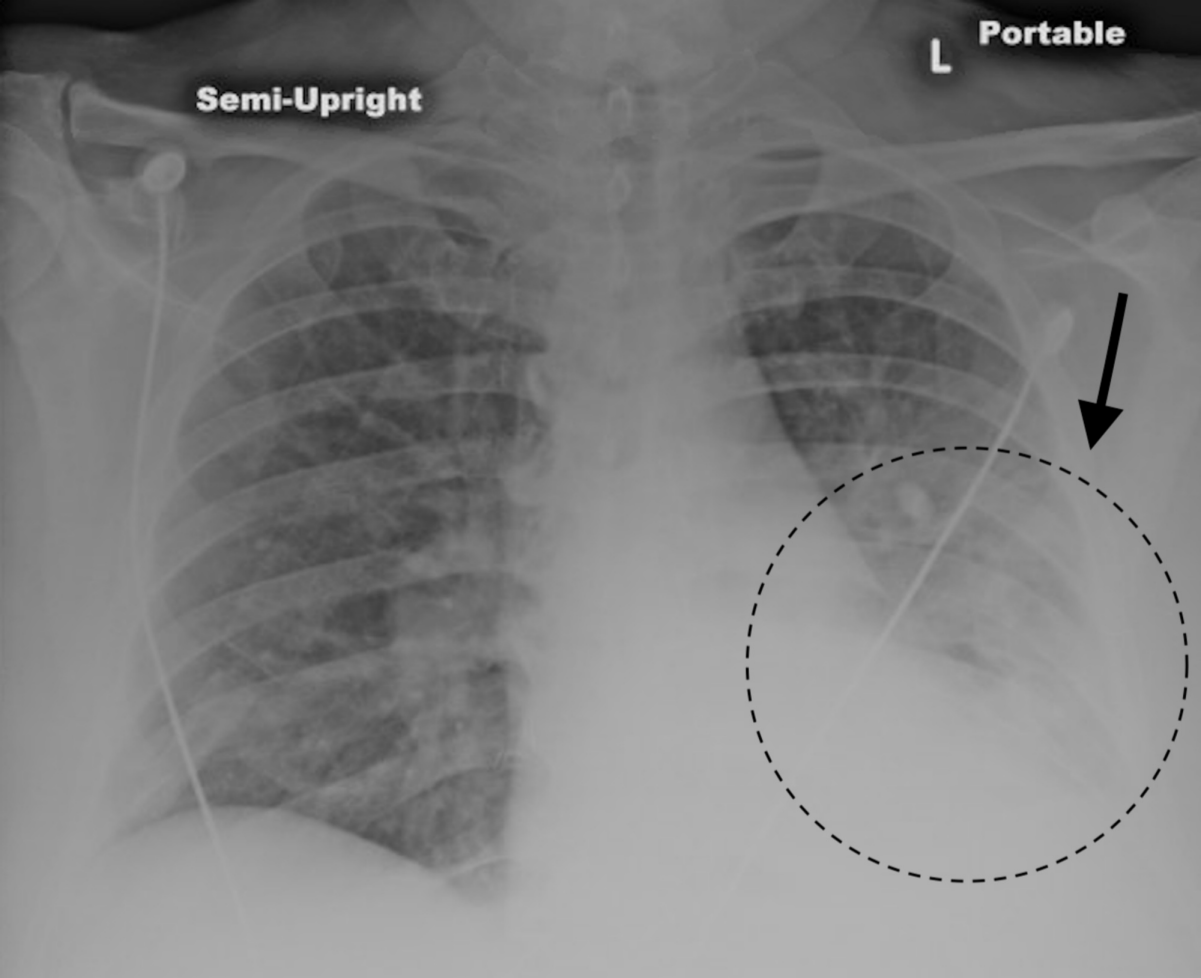
Chest x-ray on admission demonstrating a left lower lobe opacity.

A 45-year-old male with a past medical history of substance abuse, hypertension, type II diabetes mellitus, and acute pancreatitis three weeks prior to admission presented to the emergency department (ED) unresponsive and hypoglycemic. His recent acute pancreatitis was presumed secondary to alcohol use, and he was discharged to a rehabilitation facility. In the rehabilitation facility, he was found minimally responsive with a glucose fingerstick of 36 mg/dL and hypoxic. In the ED, his vitals were stable, saturating 100% on 4 liters nasal cannula (4LNC), afebrile, normal rate, and normotensive. His physical exam was only remarkable for left lower lobe (LLL) rhonchi and lethargy; his abdominal exam was benign (soft, nondistended, and nontender). Chest x-ray (CXR) and CT of the chest on day of admission showed an LLL opacity (Figure 1). His laboratory results were significant for total bilirubin of 1.6 mg/dL, alanine aminotransferase (ALT) 46 U/L, aspartate aminotransferase (AST) 65 U/L, and alkaline phosphatase (AP) of 357 U/L. His lipase and amylase were within normal limits. The next morning, he exhibited a systemic inflammatory response with tachycardia, tachypnea, and was febrile to 101.3°F without a clear source. He was started on IV vancomycin and cefepime to treat an LLL pneumonia. He began endorsing epigastric abdominal pain, back pain, and early satiety.

**Figure 2:**
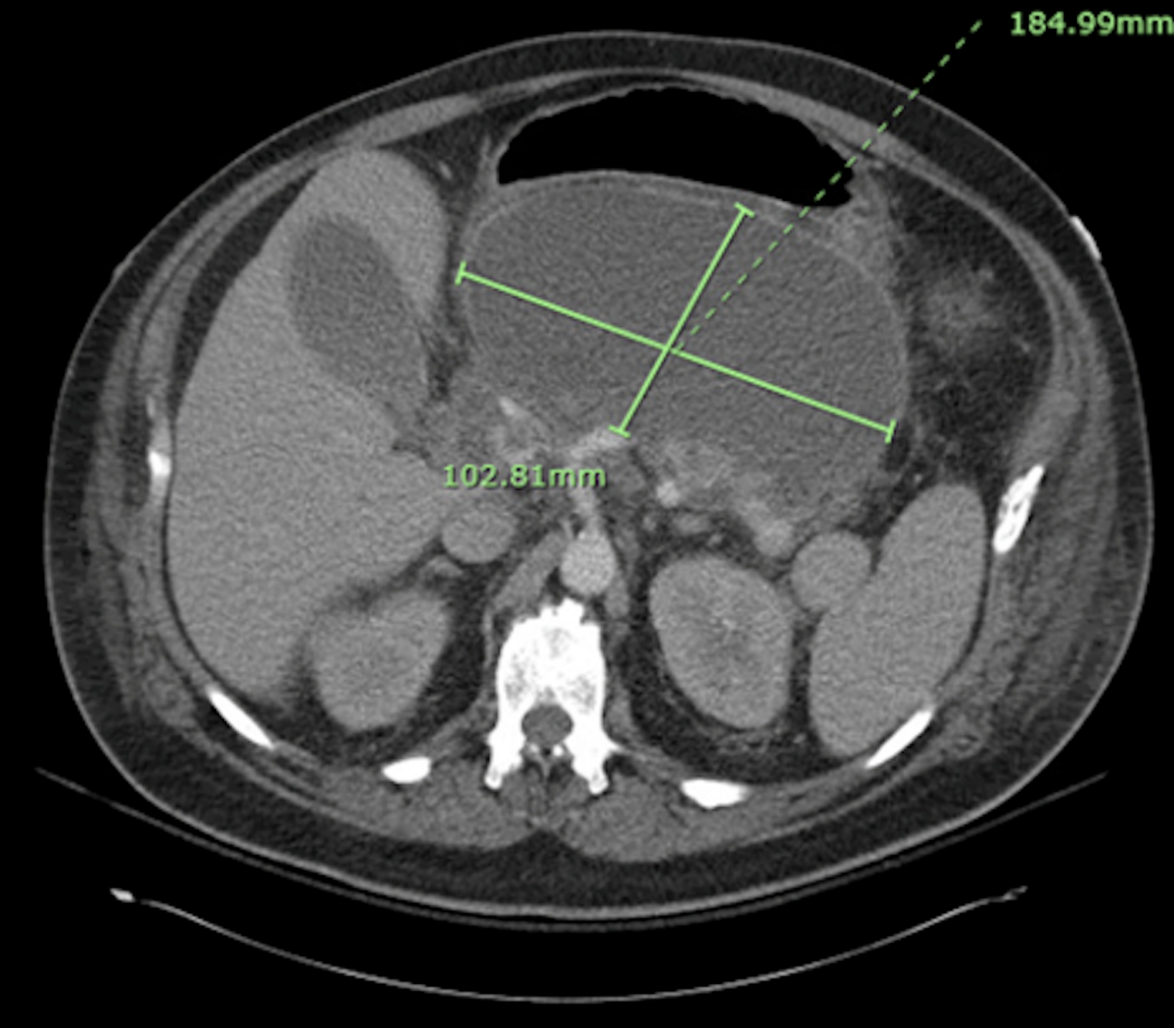
CT of the abdomen and pelvis showing an 18.5 x 10.3 cm peripancreatic cyst containing debris, most consistent with a pancreatic pseudocyst.

Thus, on day 2 he underwent CT of the abdomen and pelvis (A/P). The CT A/P demonstrated an 18.5 x 10.3 cm large cystic mass compatible with a pseudocyst containing debris, thought to be a complication from his recent pancreatitis (Figure 2) [1]. He also had a moderate left and small right pleural effusion. On day 7 of admission, his blood cultures from admission grew *Burkholderia cepacia *sensitive to ceftazidime, meropenem, and TMP-SMX. It was resistant to amikacin, cefepime, ciprofloxacin, gentamicin, and piperacillin-tazobactam. The patient was transitioned to ceftazidime. 

Due to persistent fevers on the 10th day of admission, ultrasound-guided right-sided thoracentesis was performed and 800 mL of exudative, culture negative, fluid was removed. The patient’s abdominal pain and distention persisted. He underwent CT-guided drainage of his presumed pseudocyst on day 11 and 60 mL of serous fluid was aspirated. The culture grew multidrug-resistant (MDR) *B. cepacia*, rare *Candida dubliniensis*, and rare *Candida glabrata*. Micafungin was added. *B. cepacia* had the same susceptibility pattern compared to the blood culture isolate. Despite the CT-guided drainage, the patient's clinical condition worsened. This led to a cyst gastrostomy with AXIOS stent (Boston Scientific, Marlborough, MA) placement via endoscopic ultrasound (EUS) on day 15 of admission. During the procedure, a single segmental biliary stricture was found in the lower and middle third of the main bile duct, which did not allow for a complete endoscopic retrograde cholangiopancreatography (ERCP). The drained fluid was cultured and continued to grow MDR *B. cepacia,* with the same susceptibility pattern, and its cytology was negative for malignant cells.

**Figure 3:**
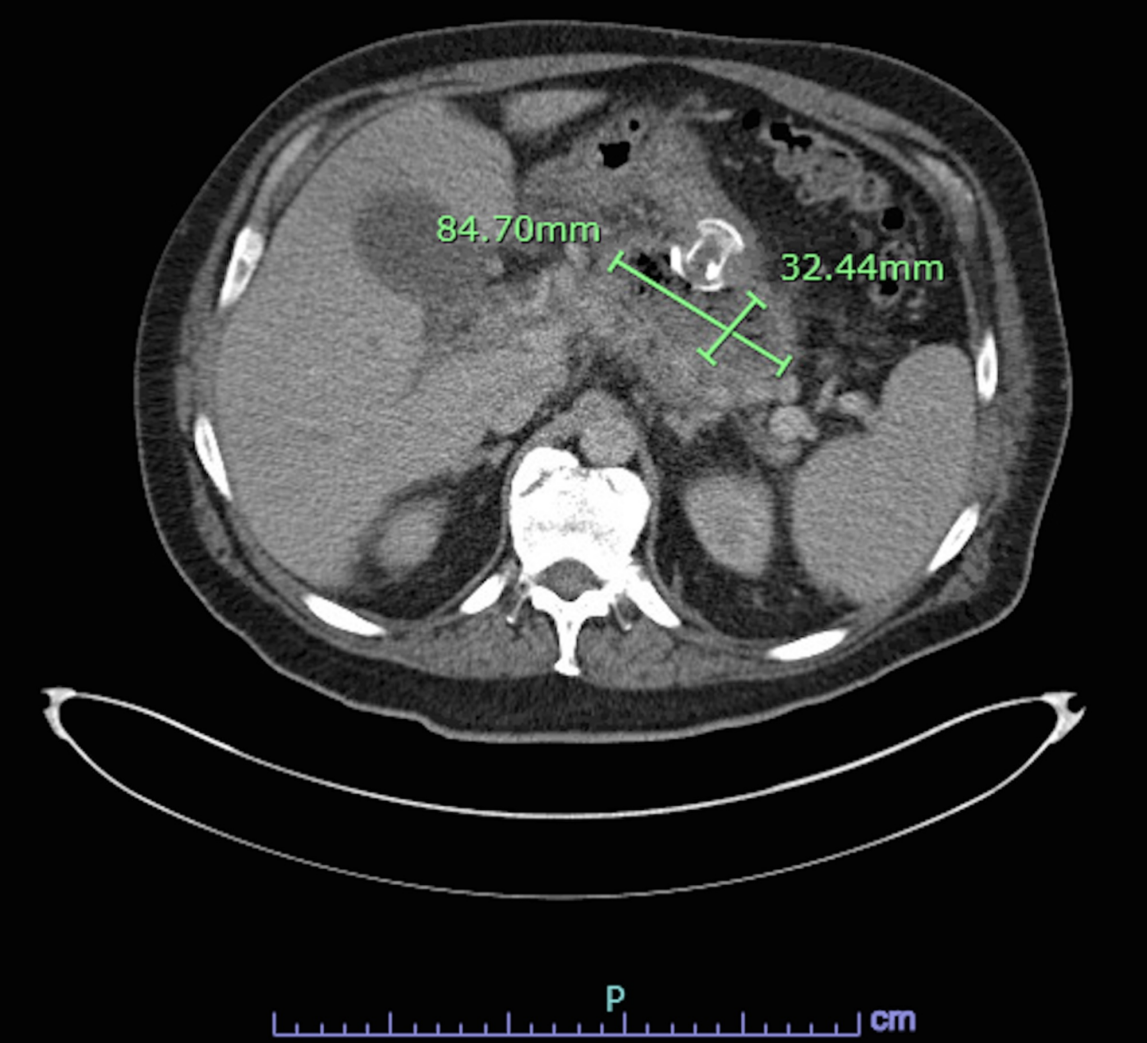
The pancreatic pseudocyst, although still present, decreased in size to 8.5 x 3.2 cm two weeks after aspiration and four weeks after initiation of antibiotics.

Two weeks later, a repeat ERCP resulted in a biliary sphincterotomy with stent placement and removal of the previously placed AXIOS stent. Repeat CT A/P two weeks after the ERCP and one month after starting antibiotics showed a decrease in the size of the cyst to 8.5 x 3.2 cm (Figure 3) [1].

Ceftazidime and micafungin were continued for a total of 10 weeks. Despite prolonged parenteral antimicrobial therapy, the patient had recurrent symptoms and growth of his pseudocyst. Four months after his diagnosis of pancreatitis, he eventually required definitive surgical intervention with exploratory laparotomy and open cyst gastrostomy. Intraoperative findings showed a large pancreatic pseudocyst filled with pus and necrotic pancreas. This finally led to a more favorable outcome with complete eradication of infection.

## Discussion

This was a unique case of a common complication of pancreatitis. Although infected pseudocysts are associated with mixed anaerobes and aerobes, *B. cepacia* has not been reported. The infected pseudocyst in this case rapidly progressed and caused severe clinical symptoms. It is important to note that the patient had persistent fevers which led to continuation of further investigation and later on, showing the need for drainage of the infected pseudocyst. It is essential to recognize that the patient’s symptoms did not completely resolve until he had adequate drainage and surgical intervention. This demonstrated that antibiotics alone are not effective in eradicating large infected pseudocysts. Moreover, the patient’s exudative pleural fluid was likely reactive secondary to the inflammatory state of the pancreas, a very common phenomenon. Instead of considering two unique processes in this patient, his sepsis from presentation could have been secondary to his infected *B. cepacia *pancreatic pseudocyst and bacteremia.

As *B. cepacia *is not a typical member of human’s gastrointestinal microbiota, we must consider iatrogenic introduction of this bacteria in our case. *B. cepacia* is typically a soil-dwelling bacterium, but is also a common colonizer in fluids in the hospital setting [9]. Interestingly, around the time of the case we present, the CDC released a report of a multistate outbreak of *Burkholderia* associated with contaminated hand foam [10]. Another epidemiologic investigation discovered 20 cases of healthcare-associated transmission of *Burkholderia* secondary to contaminated nasal spray at an ear-nose-throat (ENT) clinic [11]. After receiving treatment at two different hospitals, it is unclear how this bacterium was introduced to our patient. As far as we know, this was an isolated case, but with the bacteria isolated being multidrug resistant and with the emergence of these species, we must remain vigilant to promptly recognize patterns of contamination [12]. As this patient was immunocompetent, there was question if this patient’s intravenous drug abuse (IVDA) history had a role in acquiring this organism [1].

## Conclusions

This case shows the importance and value in including uncommon MDR organisms in the differential for infected pseudocysts for more efficient and accurate management. IVDA may be a risk factor to consider when deciding appropriate antibiotic coverage in a patient who presents with fevers and a large pancreatic pseudocyst. However, surgical intervention and/or drainage is critical for eradication of organisms in large cysts, as seen in this patient case. Future opportunities of research lie in evaluating risk factors for *B. cepacia* infections, its prevalence, and how to best manage it in unexpected sites.
